# Silencing of Long Noncoding RNA AK139328 Attenuates Ischemia/Reperfusion Injury in Mouse Livers

**DOI:** 10.1371/journal.pone.0080817

**Published:** 2013-11-27

**Authors:** Zhenzhen Chen, Shi Jia, Danhua Li, Junyan Cai, Jian Tu, Bin Geng, Youfei Guan, Qinghua Cui, Jichun Yang

**Affiliations:** 1 Department of Physiology and Pathophysiology, Peking University School of Basic Medical Sciences, Beijing, China; 2 Department of Biomedical Informatics, Peking University School of Basic Medical Sciences, Beijing, China; 3 Institute of Systems Biomedicine, Peking University, Beijing, China; 4 MOE Key Laboratory of Molecular Cardiovascular Science, Peking University, Beijing, China; Harbin Institute of Technology, China

## Abstract

Recently, increasing evidences had suggested that long noncoding RNAs (LncRNAs) are involved in a wide range of physiological and pathophysiological processes. Here we determined the LncRNA expression profile using microarray technology in mouse livers after ischemia/reperfusion treatment. Seventy one LncRNAs were upregulated, and 27 LncRNAs were downregulated in ischemia/reperfusion-treated mouse livers. Eleven of the most significantly deregulated LncRNAs were further validated by quantitative PCR assays. Among the upregulated LncRNAs confirmed by quantitative PCR assays, AK139328 exhibited the highest expression level in normal mouse livers. siRNA-mediated knockdown of hepatic AK139328 decreased plasma aminotransferase activities, and reduced necrosis area in the livers with a decrease in caspase-3 activation after ischemia/reperfusion treatment. In ischemia/reperfusion liver, knockdown of AK139328 increased survival signaling proteins including phosphorylated Akt (pAkt), glycogen synthase kinase 3 (pGSK3) and endothelial nitric oxide synthase (peNOS). Furthermore, knockdown of AK139328 also reduced macrophage infitration and inhibited NF-κB activity and inflammatory cytokines expression. In conclusion, these findings revealed that deregulated LncRNAs are involved in liver ischemia/reperfusion injury. Silencing of AK139328 ameliorated ischemia/reperfusion injury in the liver with the activation of Akt signaling pathway and inhibition of NF-κB activity. LncRNA AK139328 might be a novel target for diagnosis and treatment of liver surgery or transplantation.

## Introduction

In the past decade, one amazing finding in the analysis of the human transcriptome is that non-protein coding RNAs comprise of most transcripts in various cell tissues or cell types [1] [2]. MicroRNAs (miRNAs) are one class of small noncoding RNAs that play crucial roles in regulation of a wide range of physiological and pathophysiological process [3-6]. Long noncoding RNAs (LncRNAs), which are defined as noncoding RNA molecules greater than 200 nt in length, represent the great majority of the noncoding RNAs [7,8]. At some certain time in the development, LncRNAs can comprise of 70-90% of total RNA transcripts in the cells [9]. When LncRNAs were firstly identified, scientists believed that they may not have important biological functions [8] because of their low conservation, low expression level and high tissue specificity [8,10,11]. More recently, a number of LncRNAs had been shown to have important and diverse functions in the development and progression of various diseases [12,13]. It has been reported that LncRNAs play critical roles in the progression of cancers [14], cardiovascular diseases [2], neurodegeneration diseases [15], hepatocellular carcinoma [16,17] and focal ischemia reperfusion injury [18]. More recently, LncRNAs AB063319, AK003491 and AK044800 are shown to be abundantly expressed in brain and other tissues such as muscle, liver, lung and neuroendocrine tissues during the mouse embryonic development [19-21], suggesting that they might play important roles in regulation of mouse development. According to the LncRNA and disease database LncRNADisease [22], LncRNAs have currently been reported to be associated with more than 150 diseases. Clearly, LncRNAs are a class of molecules with important biological functions. Further intensive study of LncRNAs will shed light on the mechanisms of various diseases, and exploration of biomarkers for their diagnosis and treatment. 

Ischemia/reperfusion injury (IRI) in the liver is a major complication of haemorrhagic shock, liver surgery and transplantation, affecting millions of people worldwide each year [23]. So far, several mechanisms including adenosine triphosphate (ATP) depletion, reactive oxygen species (ROS) overproduction, macrophage activation and increase in inflammatory cytokines expression had been proposed to explain ischemia/reperfusion injury in the liver (as summarized and reviewed in references 23,24). Ischemic preconditioning has been proved effective in reducing hepatic ischemia/reperfusion injury with activation of Akt signaling pathway [25]. Pharmacological drugs that activate Akt have been shown to exhibit protective effects on ischemia/reperfusion injury in the liver [26].

 So far, there is no report regarding the role of LncRNAs in the pathogenesis of liver ischemia/reperfusion injury. In the study, we determined the LncRNA expression profile using microarray technology in the liver of mice after hepatic ischemia/reperfusion treatment. Microarray analysis revealed 98 deregulated LncRNAs in the liver after ischemia/reperfusion injury. In particular, silencing of LncRNA AK139328 attenuated ischemia/reperfusion injury in the liver with increased pAkt levels. 

## Experimental Methods

### Experimental mice and other materials

10-12 week old Male C57BL/6J mice (weight 18-20 g) were chosen in this study. All animal care and experimental protocols complied with the Animal Management Rules of the Ministry of Health of the People’s Republic of China and the guide for the Care and Use of the Laboratory Animals of the Peking University. TRIZOL reagent was from Invitrogen Inc. (NY, USA). RNeasy minicolumn was from Qiagen Inc. (Valencia, CA). GoScriptTM Reverse Transcription System and Go Taq@ qPCR Master Mix were purchased from Promega Inc (Madison, WI). Other chemicals and reagents are analytic grade. All animal protocols were approved by the Animal Research Committee of the Peking University Health Science Center.

### Hepatic ischemia/reperfusion injury model

A murine model of 70% partial hepatic ischemia was used in this study. The protocol was detailed elsewhere [27,28]. In brief, male C57BL/6 mice were anesthetized by I.P. injection of chloral hydrate (4ml/kg), and then a midline laparotomy was performed. An atraumatic artery clip was clamped at the base of left and median liver lobes to interrupt the blood supply. After 60 minutes of ischemia, the clamp was removed to initiate reperfusion. Mice were sacrificed for experimental assays after reperfusion of 6 hours. All animal protocols were approved by the Animal Research Committee of the Peking University Health Science Center.

### RNA extraction and real Time PCR assay

Liver tissues were quickly dissected from mice and snap-frozen in liquid nitrogen. Total RNA was extracted from mouse livers using Trizol reagent (Life Technologies) according to the manufacture’s recommendations. After DNase digestion, total RNA was eluted with 20 μl of RNase-free water and stored in liquid nitrogen. Purity and quantity of extracted total RNA was determined by the ratio of absorbance at A260 to A280, and argrose gel electrophoresis. Total RNA was reverse transcribed to cDNA using of the TaqMan Reverse Transcription Reagents kit (Applied Biosystems) according to the manufacturer’s protocol using random primers.

Quantitative real-time PCR was performed using the DNA Engine with Chromo 4 Detector (MJ Research, Waltham, MA). Amplification conditions were as following: 94°C for 7 minutes, followed by 35 cycles of 94°C for 30 seconds, 59°C for 30 seconds and 72°C for 30 seconds, and then final extension at 72°C for 7 minutes. The relative expression of target genes in various groups were calculated using 2^-ΔΔCt^ methodology as detailed previously [29,30]. For quantitative assay of LncRNAs in the liver, their relative expression levels were firstly normalized to house-keeping gene β-actin, and then normalized to sham or control group data values using 2^-ΔΔCt^ methodology as above [29,30]. All real time PCR products were analyzed by 1% agarose gel. All primer sequences for RT-PCR or quantitative PCR assays were listed in Table S1.

### Measurement of plasma amiotransferase activities

The activities of plasma alanine aminotransferase (ALT) and aspartate aminotransferase (AST) were determined in Department of Laboratory Medicine of Peking University Third Hospital. 

### Microarray analysis for LncRNA and mRNA

Total RNA from each sample was quantified using the NanoDrop ND-1000. The expression profiles of mouse genome-wide LncRNAs were then detected using Arraystar Mouse LncRNA Microarray v2.0, which also detected the expression profiles of mouse genome-wide protein-coding transcripts at the same time by KangChen Bio-tech (Shanghai, China). The expression profile dataset was submitted to the NCBI GEO database (GEO accession number: GSE47412). For microarray analysis, Agilent Feature Extraction software (version 11.0.1.1) was used to analyze the acquired array images. Quantile normalization and subsequent data processing were performed using the GeneSpring GX v11.5.1 software package (Agilent Technologies). After quantile normalization of the raw data, LncRNAs that at least 2 out of 2 samples have flags in Present or Marginal (“All Targets Value”) were chosen for differentially expressed LncRNAs screening. The threshold for up-regulation was fold change≥1.5 and for down-regulation, fold change≤0.7.

### Knockdown of LncRNA AK139328 in the livers of C57BL/6 mice

To knockdown hepatic AK139328 in normal C57/B6 mice, a mixture of 3 sets of stealth siRNA against mouse AK139328 (full length of mouse AK139328 sequence is available at http://www.ncbi.nlm.nih.gov/nuccore/AK139328) was synthesized by Invitrogen (sense1, 5’ CAGCUAUCACAUGCCAGCAUCAUAU -3’, antisense1, 5’- AUAUGAUGCUGGCAUGUGAUAGCUG -3’; sense2, 5’- GCAUCUAAAGCUGGUGGCAAUACUA -3’, antisense2, 5’- UAGUAUUGCCACCAGCUUUAGAUGC -3’; sense3, 5’- CCUUGGACUUCUGACUGAAUGAACU -3’, antisense3, 5’- AGUUCAUUCAGUCAGAAGUCCAAGG -3’). The siRNA mixture was administrated to C57BL/6 mice via tail vein injection at 2.5 mg/kg body weight in 100μl sterile saline as detailed previously [29]. The same amount of scrambled sequences from Invitrogen was used as a control. 72 hours later, the mice were subjected to ischemia/reperfusion treatment as above. 

### Primary hepatocyte culture

Primary mouse hepatocyte was cultured as previously described [31]. In brief, 5-6-week-old male C57BL/6 mice were anaesthetized with 10% chloral hydrate, and then catheter was placed in inferior vena cava. The liver was perfused with 1 ml of heparin (320 μ/ml), 40 ml of solution I (Krebs’s solution + 0.1 mM EGTA), and 30 ml of solution II (Krebs’s solution + 2.74 mM CaCl_2_ + 0.05% Collagenase I), respectively. The perfused liver was passed through a 400 screening size filter by flushing with RPMI 1640 medium. The hepatocytes were collected by centrifuge at 50 g for 2 minutes. Hepatocytes were re-suspended with 1640 medium and planted in 6-well plates for experiments after three washes with RPMI 1640.

### Knockdown of AK139328 in primary mouse hepatocytes

siRNAs against mice AK139328 were designed by Invitrogen.Mice primary hepatocyte with the confluence of 80% were synchronized with serum-free starvation for 24h and then transfected with siRNA (0.1nmol) or scrambled siRNA using lipofectamine 2000 (Invitrogen). Four eight hours post transfection, AK139328 level was analyzed by real time PCR.

### Histology

Paraformaldehyde-fixed, paraffin-embedded mice liver specimens sectioned at 4 μm were stained with hematoxylin and eosin for histopathological examinations using light microscopy.

### Immunohistochemical staining

In brief, liver sections were dewaxed with xylene and ethanol before rehydration. After treatment with 3% H_2_O_2_ for 8 minutes to quench endogenous peroxidase activity, sections were incubated overnight at 4 °C with primary antibody against F4/80 (Abcam, Cambridge, MA). After washing, horseradish-peroxidase-conjugated secondary antibody was added (Santa Cruz Biotechnology). After developing the color by incubation with diaminobenzidine (Zhongshan Golden Bridge, Beijing, China), sections were counterstained with hematoxylin. Cells with brown were considered to be positive for F4-80.

### Immunobloting

20-200 μg of liver or primary hepatocyte proteins were separated by 7.5%-12% SDS-PAGE, and immunobloting assay was performed as described previously [29,32]. Finally, the membrane was developed with ECL. After immunobloting assays of target proteins, the membrane was stripped with 0.2 N NaOH and reprobed for actin or other house-keeping protein as loading control. All antibodies used in this study were pruchase from Santa Cruz or Cell Signaling.

### Statistical analysis

Data were presented as mean ± S.E.M. Statistical significance of differences between groups was analyzed by *t*-test or by one-way analysis of variance (ANOVA) when more than two groups were compared.

## Results

### Determination of deregulated LncRNA profile in mouse livers after hepatic ischemia/reperfusion treatment

**Figure 1:**
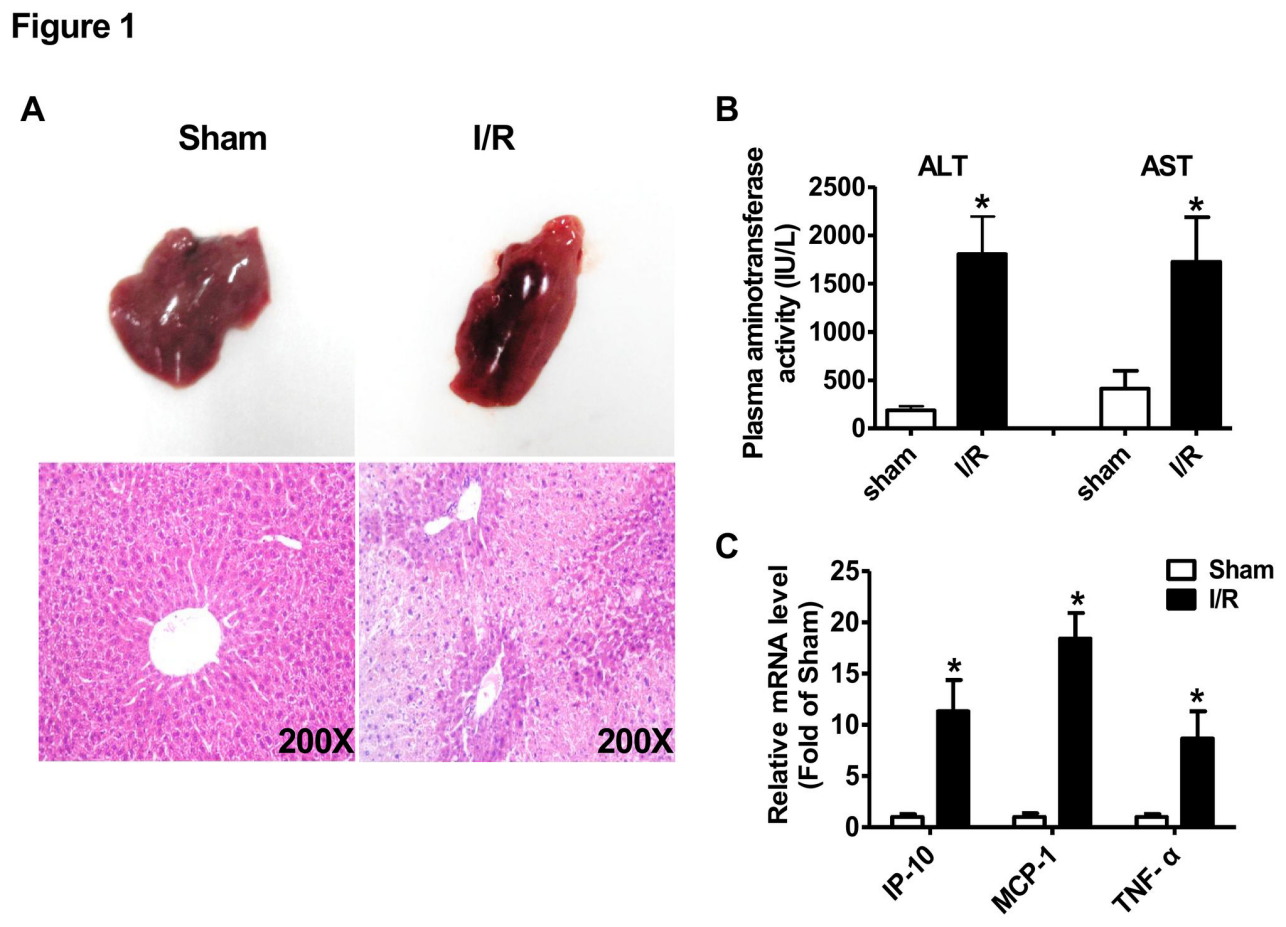
Characterics of liver injury after ischemia/reperfusion treatment. A) Liver injury indicated by morphological (upper panel) and H.E. staining (lower panel) assays. The images shown here were the representatives of at least 3 independent mouse livers. B) Plasma activities of alanine aminotransferase (ALT) and aspartate aminotransferase (AST). C) Expression of inflammatory cytokines in mouse livers after ischemia reperfusion treatment. N=5,*P<0.05 versus sham group. Sham, sham group of mice; I/R, ischemia/reperfusion group of mice.

To determine the potential role of LncRNAs in hepatic ischemia/reperfusion injury, we generated a partial hepatic ischemia/reperfusion model in mice. The ischemia/reperfusion protocol was as follows: partial (70%) hepatic ischemia for 1 hour, followed by reperfusion for 6 hours at room temperature. After 6-hour reperfusion, the mice were immediately sacrificed for liver and plasma collection. After ischemia/reperfusion treatment, morphological and H.E. staining assays revealed that liver was significantly injured (Figure 1A) with markedly elevated plasma AST and ALT activities (Figure 1B). Moreover, the expression of inflammatory cytokine IP-10, MCP-1 and TNF-α was increased in ischemia/reperfusion-injured livers when compared with sham-treated livers (Figure 1C). All these pathophysiological changes indicated the success of hepatic ischemia/reperfusion injury model in our study.

**Figure 2:**
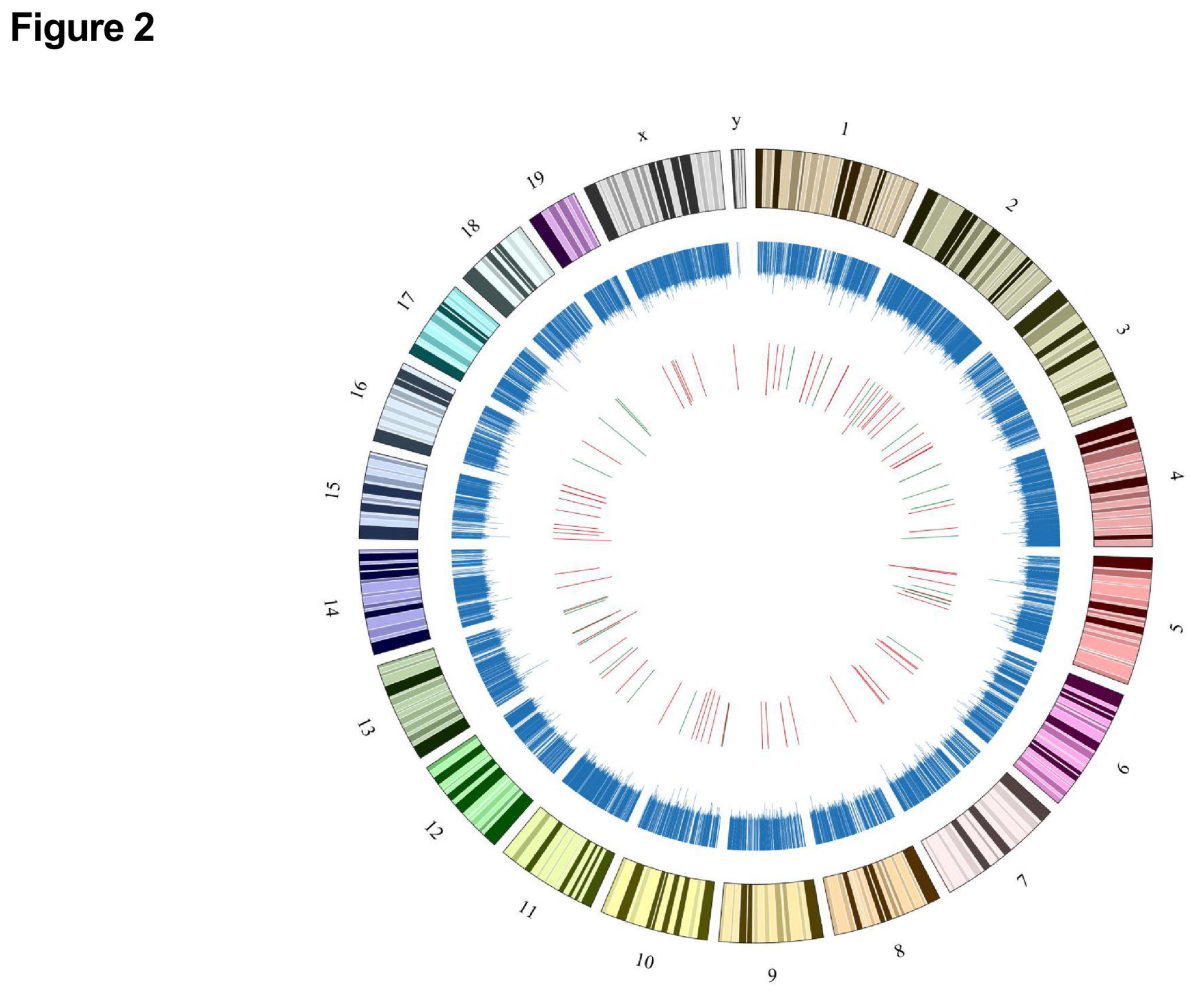
The genomic map of total lncRNAs and deregulated LncRNAs in chromosomes of mouse. The distribution of LncRNAs in chromosomes of mouse is marked in blue. The upregulated LncRNAs are highlighed in red and the downregulated lncRNAs are highlighted in green. The length of the bars represents the folds of LncRNAs expression change. Microarray assays revealed the existence of a total of 20073 LncRNAs in mouse livers. The numbers and symbols represent chromosome numbers.

**Figure 3:**
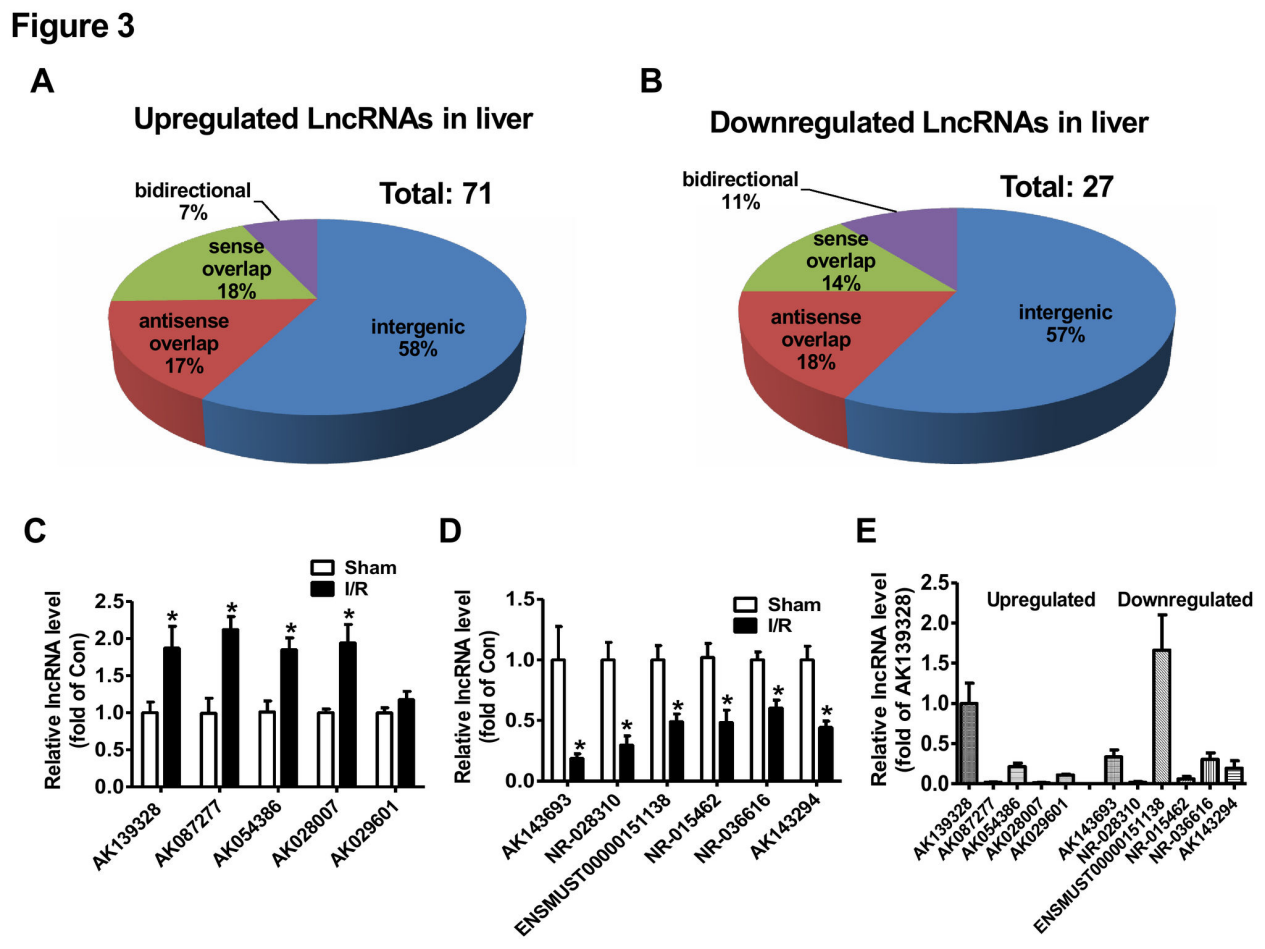
Classification and validation of deregulated LncRNAs in ischemia/reperfusion-treated mouse livers. A) Upregulated LncRNAs in mouse livers after ischemia/reperfusion treatment. B) Downregulated LncRNAs in mouse livers after ischemia/reperfusion treatment. C-D) Validation of upregulated (C) or downregulated (D) LncRNAs in the livers by real time PCR assays. E) Relative expression of LncRNAs in normal mouse livers. N=6-10, *P<0.05 versus sham group.

 The LncRNA profile in the liver of mice after hepatic ischemia/reperfusion treatment was determined by KangChen Bio-tech (Shanghai, China) using microarray technology [33]. Microarray assays revealed the existence of a total of 20073 LncRNAs in mouse livers (GEO accession number: GSE47412). The LncRNAs are ubiquitously distributed in all chromosomes including sex chromosomes of mouse (Figure 2). In ischemia/reperfusion-treated livers, 71 LncRNAs were upregulated (Figure 3A), whereas 27 LncRNAs were downregulated (Figure 3B) when compared with sham livers. The detailed information of these deregulated LncRNAs in the liver were provided in Tables S2 and S3. In ischemia/reperfusion-injured mouse livers, about 57-58% of deregulated LncRNAs are intergenic, 17-18% are antisense overlap, 14-18% are sense overlap, and 7-11% are bidirectional (Figure 3A-B). Overall, the majority of deregulated LncRNAs in the liver is intergenic. 

### Validation of deregulated LncRNAs in mouse liver after hepatic ischemia-reperfusioin treatment

To further validate the accuracy of LncRNA profile determined by microarray technology, some of the deregulated LncRNAs in the liver of hepatic ischemia/reperfusion mice were confirmed by real time PCR assay. The expression levels of five most significantly upregulated LncRNAs, AK139328, AK087277, AK054386, AK028007 and AK029601 (Table S2) were analyzed by real time PCR assay. AK139328, AK087277, AK054386 and AK028007 were confirmed to be increased, while AK029601 remained unchanged in ischemia/reperfusion-treated livers (Figure 3C). The expression levels of 6 most significantly downregulated LncRNAs, AK143693, NR-028310, ENSMUST00000151138, NR-015462, NR-036616 and AK143294 (Table S3) were analyzed by real time PCR assay. The results indicated that all of them were significantly downregulated in the liver (Figure 3D). The relative expression levels of these 11 LncRNAs in the livers of normal C57BL/6 mice were further analyzed by real time PCR assay after normalization to the mRNA level of house-keeping gene β-actin. Among these LncRNAs, AK139328 and ENSMUST00000151138 exhibited the highest expression level, whereas AK087277, AK028007, NR-028310 and NR-015462 had the lowest expression levels (Figure 3E). Overall, real time PCR assays validated the accuracy of LncRNA profile determined by microarray analysis.

### Silencing of AK139328 attenuated ischemia/reperfusion injury in the liver

**Figure 4:**
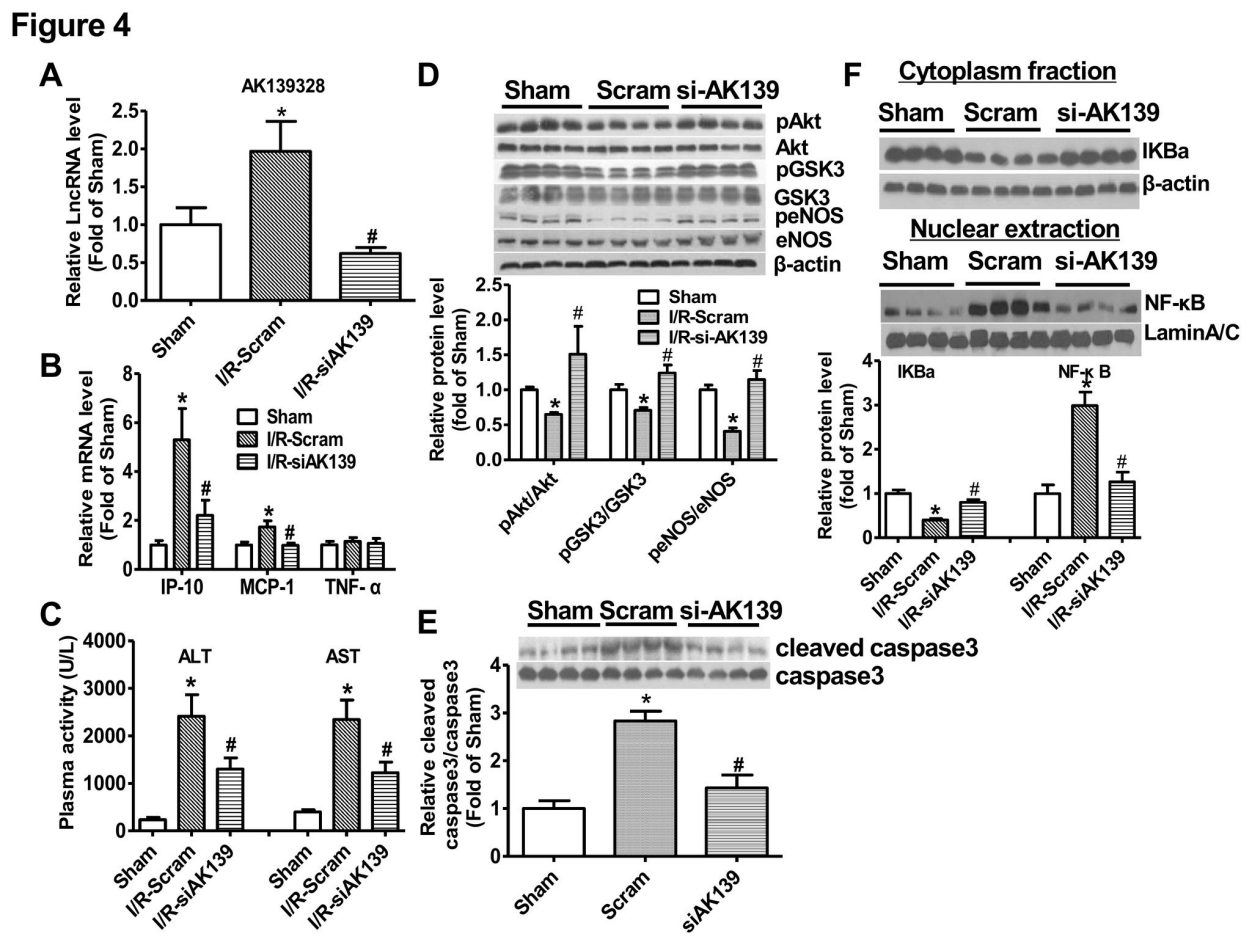
Hepatic knockdown of AK139328 on plasma activities of ALT and AST. 3 days post tail vein injection of siRNA, the mice were subjected to ischemia/reperfusion treatment. A) siAK139328 treatment reduced AK139328 level in the livers. B) Knockdown of hepatic AK139328 on the expression levels of inflammatory cytokines in the livers. C) Knockdown of hepatic AK139328 reduced plasma activities of ALT and AST. D) Knockdown of AK139328 increased posphorylated Akt (pAkt), GSK-3 (pGSK3) and eNOS (peNOS) levels in the livers. E) Knockdown of AK139328 inhibited caspase 3 activation in the livers. F) Knockdown of AK139328 inhibited NF-κB activation in the livers. The distribution of IKBa and NF-κB in cytoplasm and nuclear extraction was analyzed byimmunoblotting assays, respectively. N=8-12, *P<0.05 versus sham group, #P<0.05 versus I/R-Scramble group. Sham, sham group; I/R-Scram or Scram, scrambled siRNA treated mice; I/R-siAK139 or siAK139, siAK139328 treated mice.

Because AK139328 is one of the most significantly upregulated LncRNAs with the highest expression level among the validated LncRNAs in the liver, its role in hepatic ischemia/reperfusion injury was further evaluated. The efficacy of siRNA against LncRNA AK139328 was firstly determined in primary cultured mouse hepatocytes. The results indicated that siAK139328 treatment reduced AK139328 level by about 60% (Figure S1). siRNA against AK139328 or scrambled siRNA was injected into normal mice via tail vein. 3 days post siRNA injection, the mice were performed for hepatic ischemia/reperfusion treatment. Real time PCR assay revealed that siAK139328 injection reduced the expression level of AK139328 in the livers by about 60% when compared with scrambled siRNA-treated livers (Figure 4A). Knockdown of AK139328 reduced the mRNA levels of IP-10 and MCP-1, whereas had little effect on that of TNF-α in ischemia/reperfusion-treated livers (Figure 4B). Notably, silencing of AK139328 significantly reduced plasma activities of ALT and AST by about 50% (Figure 4C). Moreover, phosphorylated Akt (pAkt), GSK-3 (pGSK3), eNOS (peNOS) levels were increased in siAK139328-treated mouse livers when compared with control livers (Figure 4D). Silencing of AK139328 also significantly reduced cleaved caspase 3 levels in the livers, suggesting the attenuation of apoptotic pathway(Figure 4E). In the cytoplasma, IKBa level was significantly increased after silencing AK139328. Basically, IKBa binds to NF-κB and inhibits its translocation to nucleus. Consistently, silencing of AK139328 repressed nuclear translocation of NF-κB in the livers (Figure 4F). 

**Figure 5:**
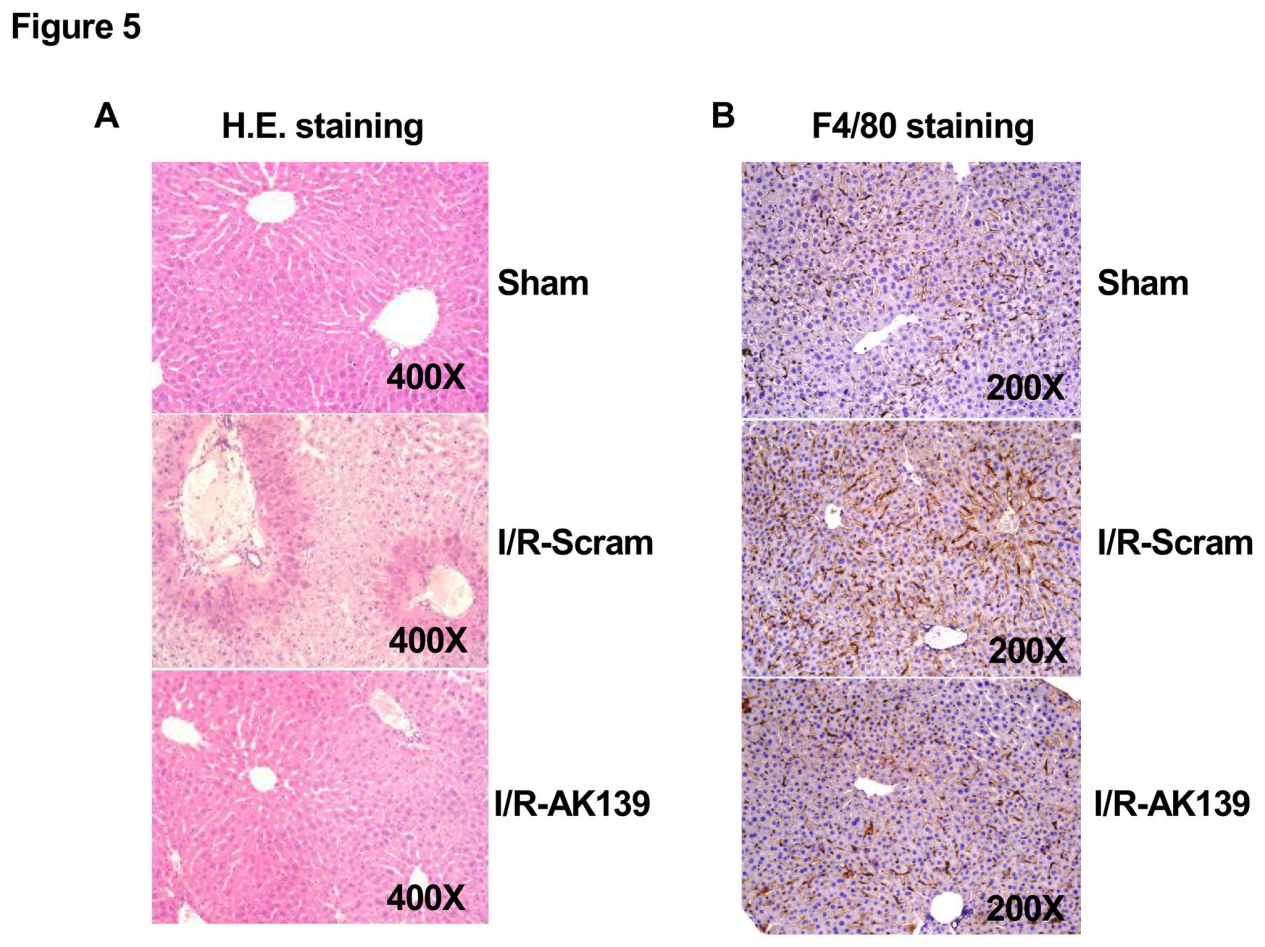
Silencing of AK139328 reduced necrosis area in ischemia/reperfusion injured livers. 3 days post tail vein injection of siRNA, the mice were subjected to ischemia/reperfusion treatment. A) Silencing of hepatic AK139328 reduced the necrosis area in the livers. The injury of liver was analyzed by H.E. staining assay. The images shown here were the representatives of 8 mice in each group. B) Silencing of hepatic AK139328 reduced macrophage activation in the livers. Immunohistochemical staining was performed using antibodies against F4/80, the biomarker for macrophage in the liver. The images shown here were the representatives of 5 mice in each group. I/R-Scram or Scram, scrambled siRNA treated mice; I/R-siAK139 or siAK139, siAK139328 treated mice.

**Figure 6:**
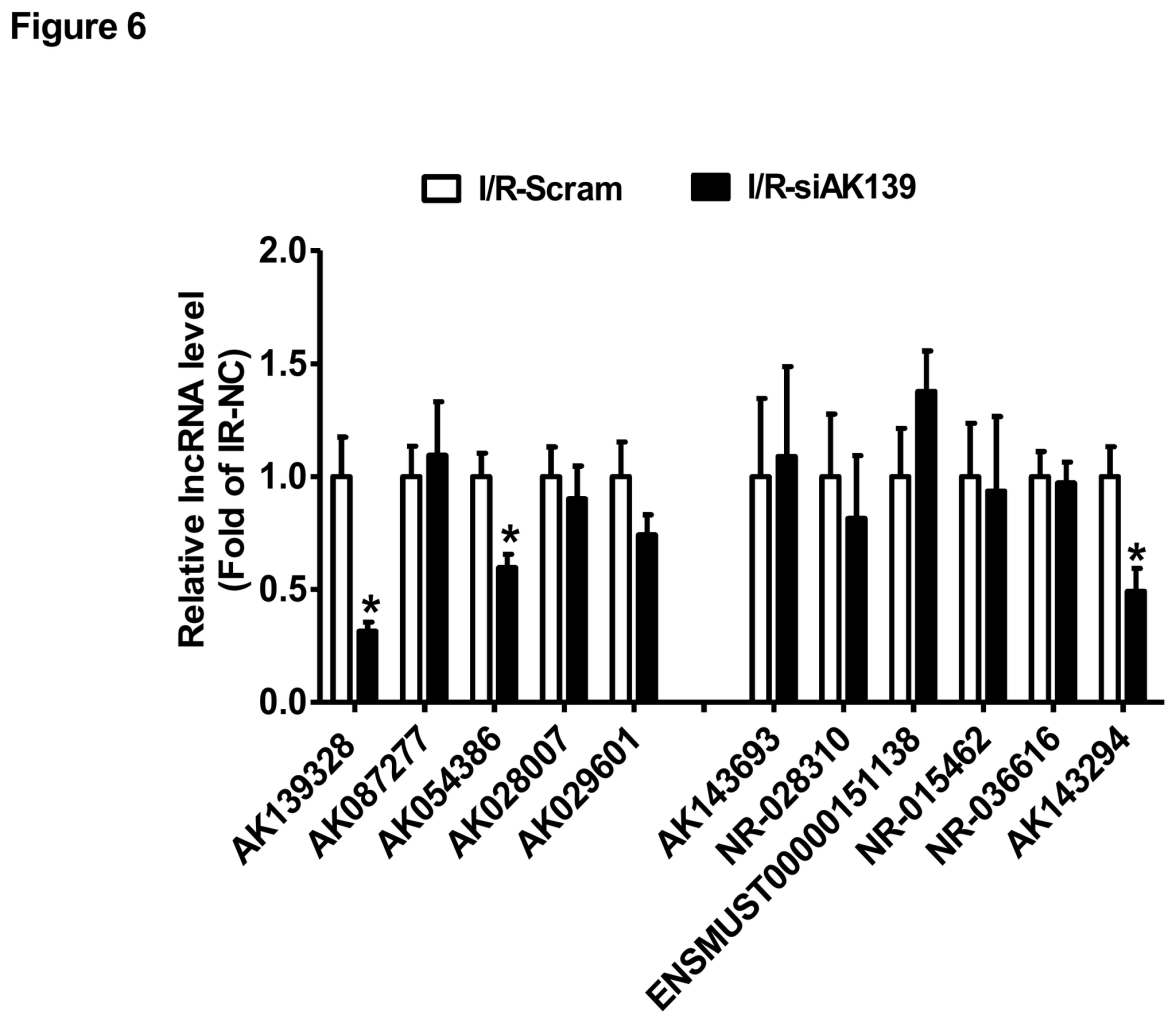
Silencing of AK139328 on LncRNA expression in the livers. 3 days post tail vein injection of siRNA against AK139328, the mice were subjected to ischemia/reperfusion treatment. The expression levels of deregulated LncRNAs shown Figure 3C/D were analyzed by real time PCR assays. N=10, *P<0.05 versus I/R-Scramble group. I/R-Scramble, scrambled siRNA treated mice; I/R-siAK139, siAK139328 treated mice.

 H.E. staining assay revealed that silencing of AK139328 significantly attenuated liver injury after ischemia/reperfusion. In siAK139328-treated livers, necrosis area was much smaller than that in control livers (Figure 5A). Immunohistochemical staining assay revealed that silencing of siAK139328 reduced the expression level of F4/80, the biomarker of liver macrophage in the livers, suggesting the inhibition of macrophage activation (Figure 5B). Among upregulated LncRNAs, the expression level of AK054386 decreased, whereas AK087277, AK028007 and AK029601 remained unchanged after hepatic silencing of AK139328. Among downregulated LncRNAs, the expression level of AK143294 was decreased, whereas AK143693, NR-028310, ENSMUST00000151138, NR-015462 and NR-036616 remained unchanged after hepatic silencing of AK139328 (Figure 6). Overall, all these pathophysiological changes suggested that silencing of LncRNA AK139328 ameliorated ischemia/reperfusion injury in mouse livers.

## Discussion

Increasing evidences have shown that LncRNAs have important biological functions and are associated with the progression of a broad range of diseases. LncRNAs are becoming novel potential molecules for disease diagnosis, treatment and prognosis. LncRNAs are shown to be involved in regulation of liver functions, in particularly in the pathogenesis of hepatocellular carcinoma (HCC). For example, LncRNA-LALR1 (An LncRNA Associated with Liver Regeneration) has been shown to accelerate hepatocyte proliferation during liver regeneration by activating Wnt/β-Catenin signaling [34]. LncRNA-Dreh can act as a tumor suppressor in the development of hepatitis B virus (HBV)-HCC [33]. LncRNA MVIH (LncRNA associated with microvascular invasion in HCC) was generally elevated in HCC, and overexpression of MVIH promotes tumor growth and intrahepatic metastasis by activating angiogenesis in mouse models [35]. In the current study, we determined the LncRNA profile in mouse livers after hepatic ischemia/reperfusion injury using microarray technology. Seventy one LncRNAs were upregulated in the liver, whereas 27 LncRNAs were downregulated in liver of mice after hepatic ischemia/reperfusion. 11 of the most significantly deregulated LncRNAs in the liver were further validated by real time PCR assays. To our knowledge, this is the first report regarding the potential role of LncRNAs in the pathogenesis of ischemia/reperfusion injury in the liver. Dharap et al identified that 359 LncRNAs were upregulated and 84 LncRNAs were downregulated at 3 hours to 12 hours of reperfusion after middle cerebral artery occlusion compared with sham group using microarray technology [18]. These studies strongly suggested that LncRNAs play important roles in ischemic-reperfusion injury in various organs.

Among the validated LncRNAs in mouse liver of hepatic ischemia/reperfusion model in our study, AK139328 exhibited the highest expression level in the normal mouse livers (Figure 3E). These findings suggest that AK139328 may play an important role in the pathogenesis of liver ischemia/reperfusion injury. To verify this hypothesis, the expression of AK139328 in mouse livers was knocked down via tail vein injection of small interfering RNA. 3 days post siRNA injection, the mice were subjected to hepatic ischemia/reperfusion treatment. Silencing of hepatic AK139328 reduced the plasma activities of aminotransferases and necrosis area in mouse livers after ischemia/reperfusion treatment. Moreover, hepatic knockdown of AK139328 also attenuated macrophage and caspase 3 activation in the livers. Clearly, these findings revealed that silencing of AK139328 attenuated ischemia/reperfusion injury in mouse livers. Mouse AK139328 is located at chromosome 2. It is an intergenic LncRNA, and is 2087114 bp at 5' side of gene encoding apoptosis inhibitor 5, and 51102421 bp at 3' side of gene encoding leucine-rich repeat-containing protein 4C precursor. The size of AK139328 is 2670 bp according to the database in NCBI (http://www.ncbi.nlm.nih.gov/nuccore/AK139328). The mRNA levels of leucine-rich repeat-containing protein 4C precursor and apoptosis inhibitor 5 were not significantly changed in ischemia/reperfusion treated livers when compared with control livers (data not shown), suggesting that AK139328 had no direct relationship with the expression of these two genes.

 In the past decades, it had been widely accepted that Akt, also known as protein kinase B (PKB), functions as a survival kinase by phosphorylating a number of apoptosis-related molecules such as BAD, forkhead transcription factors, caspase 9, and IkappaB kinase and GSK-3β. Akt's broad scope places it at the center of multiple signaling pathways, allowing it to play a protective role in various organs affected by ischemia/reperfusion injury [36]. Activation of Akt has been shown to protect against liver ischemia/reperfusion injury [25,26,37]. Silencing of AK139328 elevated pAkt level in mouse livers after hepatic ischemia/reperfusion, suggesting it may be involved in regulation of Akt activity in liver cells. In support, LncRNA urothelial carcinoma associated 1 (UCA1) regulates cell cycle distribution via CREB through PI3K-AKt dependent pathway in bladder carcinoma cells. Knockdown of UCA1 increased Akt expression and activity in bladder carcinoma cells [38]. LncRNA steroid receptor RNA activator (SRA) can enhance adipogenesis and adipocyte function through multiple pathways including Akt activation [39]. Overall, these findings suggested that LncRNAs may be widely involved in regulation of Akt activation in various cell types. As one main downstream molecule of Akt, GSK-3 plays an important role in liver ischemia/reperfusion injury. Inhibition of GSK-3 has been shown to ameliorate liver ischemia/reperfusion injury [40-42]. In this study, we found that silencing of AK139328 resulted in inhibition of GSK-3 in ischemia/reperfusion treated mouse livers, which is consistent with Akt activation. Activation of eNOS has been reported to ameliorate ischemia/reperfusion injury in the liver [43]. Moreover, it has been further reported that activation of Akt ameliorates liver ischemia/reperfusion injury via activation of eNOS-NO-HIF pathway [44]. In this study, we found that silencing of AK139328 significantly increased pAkt and peNOS levels, and protect against hepatocyte death in ischemia/reperfusion livers.

 Activation of nuclear factor κB (NF-κB) also plays an important role in liver ischemia/reperfusion injury by regulating the expression of proinflammatory cytokines [45]. Inactivation of NF-κB has been shown to protect against hepatic ischemia/reperfusion injury [46,47]. In support, the protective effects of xanthohumol and triiodothyronines on liver ischemia/reperfusion injury have been reported to be associated with reduced NF-κB activity [48,49]. In this study, we found that silencing of AK139328 ameliorated liver injury with inhbition of NF-κB activity and inflammatory cytokine expression. However, it has also been reported that activation of NF-κB is protective in hepatic ischemia/reperfusion injury [50,51]. Overall, the protective effects of AK139328 silencing in hepatic ischemia/reperfusion injury were associated with activation of Akt and repression of NF-κB activity.

 As summarized and reviewed in reference [9], LncRNAs can function as cis-tether, cis-targeting, trans-targeting, enhancer, decoy, scaffold, allosteric modification, co-activator or co-repressor to regulate gene expression. To identify the mechanism through which LncRNA AK139328 mediates Akt activation in liver cells will shed light on the mechanisms of liver ischemia/reperfusion injury. Moreover, further study on the crosstalks among AK139328 and other deregulated LncRNAs will also provide insight into understanding the roles of LncRNAs in liver ischemia/reperfusion injury. 

In conclusion, we presented the first report exploring the LncRNA expression profile in mouse liver after hepatic ischemia/reperfusion treatment. A total of 98 LncRNAs were deregulated in the liver of mice after hepatic ischemia/reperfusion treatment. Silencing of LncRNA AK13328 attenuated ischemia/reperfusion injury in the liver, suggesting that it might be a novel target for treatment of ischemia/reperfusion injury when liver surgery or transplantation is performed.

## Supporting Information

Table S1 **List of oligonucleotide primer pairs used in real time RT-PCR and RT-PCR analysis.**
(DOC)

Table S2 **The characterics of upregulated LncRNAs in Ischemia/Reperfusion injured mouse livers.**
(XLS)

Table S3 **The characterics of downregulated LncRNAs in Ischemia/Reperfusion injured mouse livers.**
(XLS)

Figure S1 **The effect of siAK139328 treatment on AK139328 level in mouse hepatocytes.** The cultured hepatocytes were transfected with siAK139328 or scrambled siRNA as described above. Fourty eight hours post siRNA transfection, AK139328 level was analyzed by real time PCR assay. N=5, **P<0.01 versus Cells treated with Scrambled siRNA.
(TIF)
