# Supplementary material for: Silencing of Long Noncoding RNA AK139328 Attenuates Ischemia/Reperfusion Injury in Mouse Livers
Source: PLoS One. 2013 Nov 27;8(11):e80817. doi: 10.1371/journal.pone.0080817 (PMC3842297; doi:10.1371/journal.pone.0080817)
Supplement: Table S1 — List of oligonucleotide primer pairs used in real time RT-PCR and RT-PCR analysis. (DOC) [file pone.0080817.s001.doc]

Table S1. List of oligonucleotide primer pairs used in real time RT-PCR and RT-PCR analysis.

| LncRNA or Gene ID | Sense Primer(5'-3') | Antisense Primer(5'-3') | Annealing temperature |
| --- | --- | --- | --- |
| AK087277（M） | ccctggaaaacacaggaaaa | gaagacggagaagccacaag | 59℃ |
| AK028007(M) | ggaacagcgtttgaaaggtc | gggatctccctcttctcagg | 59℃ |
| AK139328 (M) | ccagttcttggtcctggtgt | gtgtctgcaacccgataggt | 59℃ |
| AK054386 (M) | atcgctgtttggatttcctg | attatggtgctgggctgaac | 59℃ |
| AK029601(M) | caagcaaccatttgacgaga | cccctggaccctttctatgt | 59℃ |
| ENSMUST00000151138 (M) | ACTTCCCCTCTGCAGTCTGA | CTCACAGAGCCATCCTGACA | 59℃ |
| NR-028310(M) | aagccaaaccgacagaaatg | acgttcaccaacacctctcc | 59℃ |
| NR-015462(M) | ataccctggacatgctggag | gatgagcagtgggtggctat | 59℃ |
| AK143294(M) | ctacaagccccatcaccact | gctttcaccagatcccaaaa | 59℃ |
| NR-036616(M) | tgctctgcgtaaactggatg | agggcctgaaaggagagaag | 59℃ |
| AK143693（M） | gggtccacctctctgtcaaa | AAGCACTTCCGAGGTTCAGA | 59℃ |
| IP-10（M） | AAGTGCTGCCGTCATTTTCT | GTGGCAATGATCTCAACACG | 59℃ |
| TNF-a（M） | CACAAGATGCTGGGACAGTGA | TCCTTGATGGTGGTGCATGA | 59℃ |
| MCP-1（M） | AATGAGTAGCAGCAGGTGAGTG | GAAGCCAGCTCTCTCTTCCTC | 59℃ |
| GAPDH（M） | aactttggcattgtggaagg | acacattgggggtaggaaca | 59℃ |
| β-actin (M) | AGCCATGTACGTAGCCATCC | GCTGTGGTGGTGAAGCTGTA | 59℃ |

M: mouse. If not indicated, all the primer sequences are referred to mouse origin.
