# Supplementary figures and images for: Silencing of Long Noncoding RNA AK139328 Attenuates Ischemia/Reperfusion Injury in Mouse Livers
Source: PLoS One. 2013 Nov 27;8(11):e80817. doi: 10.1371/journal.pone.0080817 (PMC3842297; doi:10.1371/journal.pone.0080817)

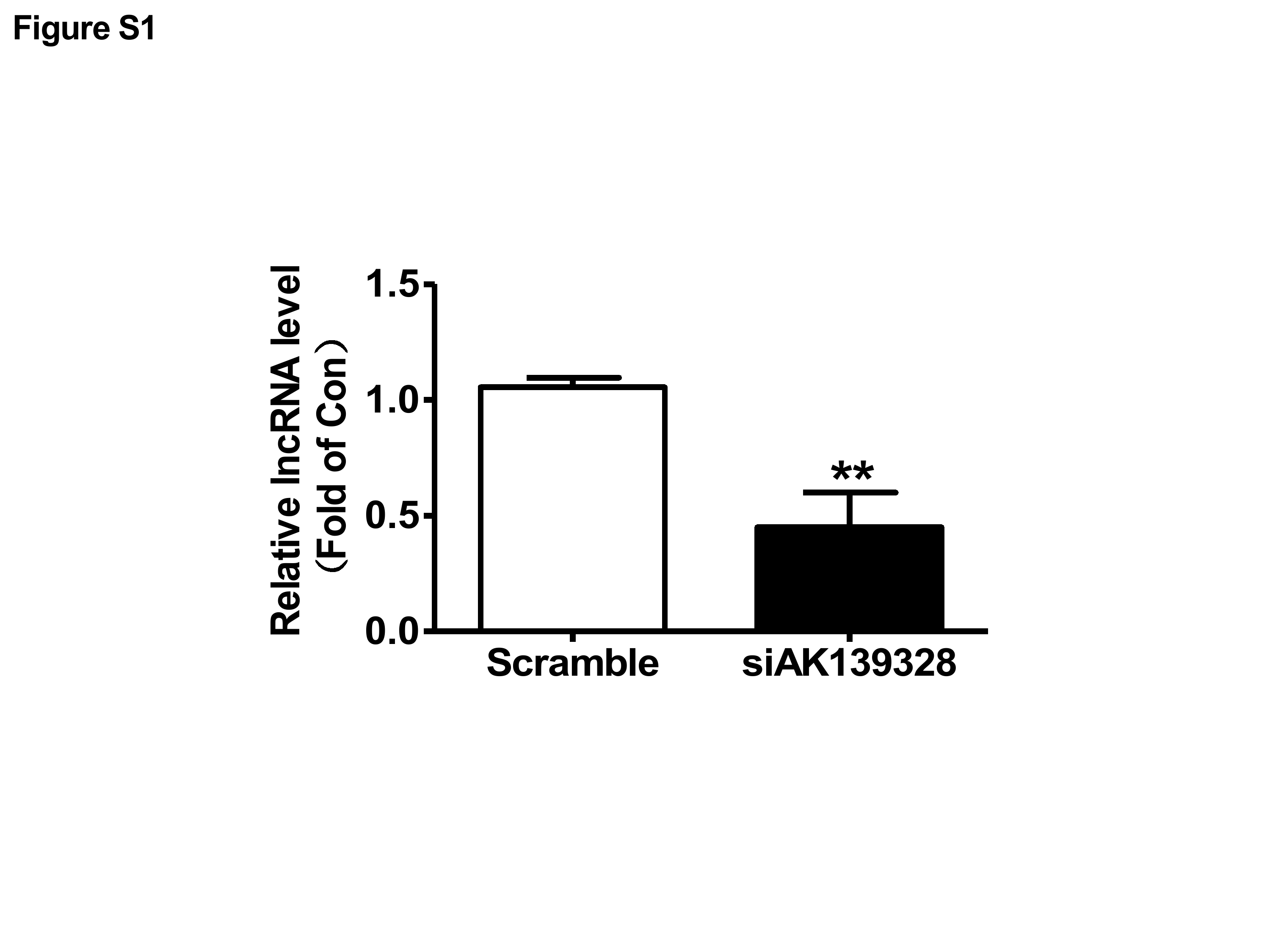

Supplement: Figure S1 — The effect of siAK139328 treatment on AK139328 level in mouse hepatocytes. The cultured hepatocytes were transfected with siAK139328 or scrambled siRNA as described above. Fourty eight hours post siRNA transfection, AK139328 level was analyzed by real time PCR assay. N=5, **P<0.01 versus Cells treated with Scrambled siRNA. (TIF) [file pone.0080817.s004.tif]
